# Deconstructing the “types” of osteoarthritis

**DOI:** 10.1016/j.ostima.2024.100257

**Published:** 2024-12-18

**Authors:** David J. Hunter, Leticia A. Deveza

**Affiliations:** a Sydney Musculoskeletal Health, Arabanoo Precinct, Kolling Institute, Faculty of Medicine and Health, The University of Sydney, Sydney 2065 NSW, Australia; b Rheumatology Department, Royal North Shore Hospital, St Leonards, NSW 2065 Australia; c Northern Clinical School, Faculty of Medicine and Health, The University of Sydney, Australia; d Clinical Research Centre, Zhujiang Hospital, Southern Medical University, Guangzhou, China

**Keywords:** Phenotype, Endotype, Theratype

## Abstract

The community acknowledges the staggering prevalence and disabling nature of osteoarthritis, and the crucial need for therapeutic advancement. In our quest to define a clinically meaningful endpoint and identify biomarkers that can serve as short-term treatment responses and reliable predictors of long-term outcomes, we must also strive to target therapies more effectively. This perspective article not only aims to elucidate the nomenclature of osteoarthritis types but also proposes a path towards greater transparency that has the potential to inspire a new era of both research and clinical practice.

## Introduction

For a long time, our community has discussed the notion of a phenotype. This emanates from the long-standing acceptance of osteoarthritis heterogeneity. Much of the literature on phenotypes is driven by hypothesis generation and theory, which largely remain to be tested by true hypothesis testing, data-driven exercises with external validation in independent samples.

Although a disease is usually defined as an entity associated with a specific cause and specific anatomical or functional abnormalities, a syndrome, such as OA, is characterized by similar signs and symptoms with different causes and manifestations [1]. Knowledge of OA “types” will help to unpack our umbrella disorder (complex syndrome) into more well-defined subgroups of people living with osteoarthritis. These would likely encompass a number of typical pain and pathobiological mechanisms [2,3]. This perspective article aims to provide a platform for the phenotype in osteoarthritis and the evolution of other nomenclature, including the endotype and theratype as they relate to OA.

## Deconstructing the phenotype

The term 'phenotype' is often used loosely, and it's important to clarify its meaning in the context of osteoarthritis. A phenotype refers to a composite of observable characteristics or traits of an individual that results from genetic and environmental factors [4]. In the case of osteoarthritis, each phenotype should focus on distinct pathobiological or pain mechanisms [2]. While it's possible for individuals with osteoarthritis to have overlapping phenotypes, a clear description should allow for the delineation of these distinct mechanisms. By using a consistent framework, we can facilitate standardization in meta-analyses and other comparative data exercises, encouraging a more unified approach to research and treatment.

A systematic review of osteoarthritis phenotypes [5] identified six phenotypes that included 1) chronic pain phenotype with central sensitisation; 2) inflammatory phenotype; 3) metabolic syndrome phenotype; 4) bone and cartilage metabolism phenotype; 5) mechanical (malalignment) phenotype; and 6) minimal joint disease phenotype. As mentioned earlier, much of this emanates from theory and needs to be adequately tested. This knowledge of phenotypes could be used to provide patient communities with knowledge about their prognosis, for clinicians to facilitate tailoring treatments [6], or for unravelling pathogenesis [7], and/ or providing insights into targets for novel therapies [8].

One long-term goal here is to apply this knowledge in clinical practice. As mentioned, this could be related to the prognosis or, alternatively, the likelihood of responding to a particular type of therapy. Deconstructing the clinical encounter to inform on prognosis and treatment is likely to be a complex exercise, and decision tools to facilitate translation into clinical practice will likely be required. The analogy here is to the use of FRAX in osteoporosis for prognostication as it relates to fracture risk. This was complicated to develop, and its use in clinical practice is facilitated by online algorithms. We need to develop similar tools in osteoarthritis to inform both disease risk and prognostic information as it relates to the likelihood of progression. These decision tools will likely incorporate information from a person's demographic characteristics and clinical information, such as patient-reported outcomes, in addition to potentially incorporating detailed biochemical biomarkers, imaging and omic data. The emergence of electronic medical records and bioinformatics approaches to large datasets may facilitate the emergence of these methods and the application of these methodologies in clinical practice.

## Elucidating the endotype

Leaping from the phenotype platform, if we are able to identify specific subgroups of people, it will facilitate the emergence of endotypes [9]. This is a subtype of disease defined functionally and pathologically by a molecular mechanism [4]. This knowledge of disease mechanisms will explain the disease expression in this group of patients. It is highly unlikely that one pathomechanism will be at play for each individual patient in our complex disease. Like most other common noncommunicable diseases, osteoarthritis is likely to have multiple genetic and environmental factors that increase risk and portend to disease progression. These endotypes may include mechanisms and markers related to senescent cells in ageing or cell senescence, IL-1/ hsCRP for an inflammatory type, adipokines or HbAIC for metabolic typing, or sex hormone levels for hormonal dysregulation.

## Introducing the theratype

Elucidating disease mechanisms and identifying markers that may facilitate stratification of individuals where that mechanism could be targeted allows us to introduce the “theratype”. A theratype is defined as the prediction of treatment response based on a particular disease endotype.

Results from RCTs generally reflect the average treatment effect in the study population, which does not take into account heterogeneous responses to treatment across the patients [1]. If a particular subgroup of patients experiences benefits from a given treatment but another subgroup does not, the trial's result may be largely negative if the subgroup with a positive response is not identified properly.

An example of this theratype methodology that emerges from our field is the application of PRO-C2 in the sprifermin FORWARD study [10]. Whilst a posthoc analysis, it demonstrated that participants with lower levels of type 2 collagen formation appeared more responsive to this anabolic agent. An analogous approach might be used in the context of a trial targeted towards an inflammatory phenotype, such as an IL-1 inhibitor. One might use a particular biomarker at baseline screening, such as contrast-enhanced MRI of synovitis or Doppler inflammation on ultrasound, to identify a population more amenable to this intervention. These could be used in a predictive context of use to enrich participant populations in clinical trials [11]. Similar approaches have emerged from the application of machine learning to find subgroups based on baseline characteristics ([12,13]).

This context of use is not to be confused with another critically important need in our field: determining an intervention's efficacy via a pharmacodynamic/ response biomarker. A recent example is the use of serum ARGS-aggrecan in the ROCCELLA trial, testing the anti-catabolic ADAMTS-5 inhibitor (S201086/ GLPG1972) [14]. The intent was to identify a population of people where altered ADAMTS-5 activity may have portended treatment success from this particular therapy. Unfortunately, it didn't play out that way, and while the drug lowered serum ARGS, this reduction didn't flow into an improvement in either patient-reported outcomes or MRI cartilage thickness. This methodology is analogous to that utilised some years ago in the risedronate trial, where reductions in CTX-II at 6 months with treatment were associated with radiological progression at 24 months [15].

## Outlook

**Fig. 1:**
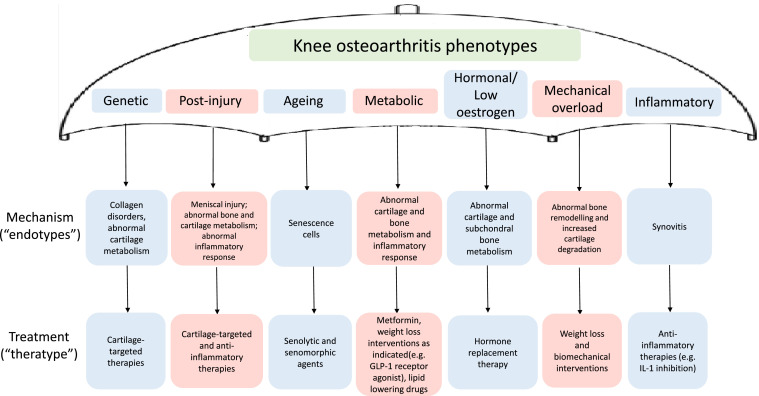
Example of how the concept of pheno-/endo-/theratyping can be used to maximize treatment success in knee osteoarthritis, noting that phenotypes may not be exclusive so different treatment types (or combination of treatments) may be suitable to the same patient.

This is an emerging field and the methods utilised thus far need further maturation. Phenotypes and endotypes will require appropriate validation in independent datasets. Theratypes will continue to emerge, and ideally, novel clinical trial designs will incorporate some knowledge of mechanisms into population stratification and assessment to see enrich study samples for this most amenable to particular mechanistic targets. The accompanying Fig. 1 provides a schematic to assist in deconstructing this “type” concept. For each person with osteoarthritis, they may be affected by more than one phenotype at the same time. In addition, for each endotype, multiple different mechanisms may be operational at any one time. Consequent to that, each theratype may have more than one mechanistic target. It is difficult to convey this complexity and in reality, there would likely be multiple lines between phenotypes, endotypes and theratypes.

If one were to adopt an approach using endotypes to stratify a population that was likely more amenable to a particular mechanistic target, there are a number of implications worthy of further consideration. The most immediate advantage of adopting this methodologic approach would be to optimise an opportunity to demonstrate efficacy by recruiting a population that is likely amenable. The greatest disadvantages would likely be increasing markedly the screen failure rate and the external validity of these findings. It is this latter concern about generalisability that regulators may be most concerned about, and they might limit the product label to this particular population.

The increasing availability of longitudinal, high-quality data from both observational datasets and clinical trials is facilitating the examination of these methods. Given the complexity of these datasets and the importance of not being wedded to old theories, bioinformatics approaches with the application of machine learning methodologies and deep learning can facilitate further advances.

Recognition of the availability of this data in our field has led to efforts to perform methodologically sound analyses to assess the efficacy of several different interventions in predetermined subgroups of patients who might have a better response to a given treatment. This is the focus of the osteoarthritis trial bank [16], which provides a unique opportunity to combine data from many trials. This methodology will be applied in the near future to disease-modifying therapies in osteoarthritis. This proposed approach, working under the auspices of the Foundation of National Institute of Health (FNIH) [17], will harvest extant DMOAD trials and apply individual patient data and machine-learning approaches to identify subgroups of patients and markers that facilitate their stratification.

The promise for our field is great. If we are to truly emerge from this era of precision medicine with targeted therapies for osteoarthritis, we need to apply this knowledge both to our existing datasets and prospective clinical trials. Our obligation is for our patients to meet the disease burden with answers that accelerate the field. The application of knowledge through decision aids will hopefully go some way to addressing their needs. Accelerating therapeutic development will further enhance that goal.

## Declaration competing of interest

DJH is employed by the University of Sydney and Royal North Shore Hospital. His salary support for the University of Sydney is supported by Arthritis Australia and an NHMRC Investigator Grant Leadership 2 (#1,194,737).

DJH is the editor of the osteoarthritis section for UpToDate and co-Editor in Chief of Osteoarthritis and Cartilage.

DJH provides consulting advice on scientific advisory boards for Haleon, TLCBio, Novartis, Tissuegene, Sanofi, Enlivex.

LAD receives royalties from UpToDate.
